# Stigma of addiction and mental illness in healthcare: The case of patients’ experiences in dental settings

**DOI:** 10.1371/journal.pone.0177388

**Published:** 2017-05-22

**Authors:** Mario A. Brondani, Rana Alan, Leeann Donnelly

**Affiliations:** 1 Faculty of Dentistry, University of British Columbia, Vancouver, British Columbia, Canada; 2 Henry M. Goldman School of Dental Medicine, Boston University, Boston, Massachusetts, United States of America; University of Washington, UNITED STATES

## Abstract

**Objective:**

To explore the ways in which stigma is experienced in healthcare and dental settings by patients with a history of addiction and mental illness.

**Methods:**

Audio-recorded, semi-structured interviews with a purposefully selected convenience sample of residents from two community treatment centres in Vancouver, Canada were conducted. The interview guide contained questions about experiences while seeking health and dental care and was based on an existing framework of labeling, stereotyping, exclusion, discrimination, and power imbalance. Interviews were transcribed *verbatim* for coding and thematic analysis.

**Results:**

Twenty-five participants between 23 and 67 years of age were interviewed; 17 were males. Most had a self-reported history of depression combined with use of alcohol and crack-cocaine; most of them only sought dental care for emergency purposes. Textual analysis of more than 300 pages of transcribed interviews revealed that participants perceived stigma when they were negatively stereotyped as ‘unworthy’, labeled as ‘different’, excluded from the decision-making process, discriminated against, ‘treated unfairly’, and felt powerless when interacting in the heath and dental care systems. Conversely, positive experiences were characterized by empathy, reassurance and good communication, which were empowering for patients.

**Conclusions:**

When associated with stigma, mental illness and addictions have negative implications for accessing health and dental care. From our participants’ perspectives, it seems that the lack of understanding about their life conditions by the healthcare professionals was the origin of stigma. We suggest that an increased social awareness of these health issues be enhanced among current and future health and dental care professionals to help improve care experiences for this marginalized population.

## Introduction

The feeling of being stigmatized is a reality for many individuals because of the way they look, behave or present themselves to society; it remains a barrier to their well-being. The term stigma originates from ancient Greece, referring to a symbol in the form of cut or burnt into certain peoples’ skin to show their moral deficiency [1]. According to Smith (2011), stigma is a deeply discrediting attribute [1], while the process of stigmatization can be conceptualized as someone being labeled, stereotyped, socially excluded, and discriminated against [2]. Stigmatization undeniably occurs through the exercise of power as suggested by Link and Phelan in 2001 [2], while other conceptualizations and portrayals of stigma exist, from Goffman’s deeply discrediting attribute and social disqualification [3] to Thornicroft’s combination of ignorance, prejudice and discrimination that may lead to negative attitudes, rejection and avoidance [4] as discussed by Alan in 2014 [5].

Stigma associated with comorbidities such as addiction and mental illness is often held by healthcare providers [6], and by the population at-large [7]. In North America in general, and in Canada in particular, it is estimated that most people drink alcohol and close to half of them have used substances such as cannabis, hallucinogens, cocaine, amphetamines, ecstasy, heroin, steroids, lysergic acid diethylamide, and inhalants at least once during their lifetime [8]. A recent survey has shown that in Canada 16% of people who do develop substance addiction also have co-occurring mental illness [9].

Given that one in every six individuals who have a history of addiction also experience mental illness [9], the presence of stigma may cause delay or avoidance in seeking health care treatment including dental care. Such avoidance worsens disease progression, reduces treatment compliance, and further increases the risk of relapse. It also worsens oral diseases that most substance users suffer from including dental decay and abscesses [10,11] while contributing further to the inverse care law as those with the greatest needs face considerable challenges in getting proper treatment [11].

Although the literature on stigma and healthcare in general is substantial, there are a lack of studies focused on stigma faced by individuals with a history of addiction and mental illness when accessing dental care in particular. For example, Charnock and colleagues in 2004 explored dental attendance of drug users and found that while the majority of them reported problems with their teeth, 25% had never visited a dentist and almost half of them did only when in pain [12]. Even though underutilization of dental services can be influenced by various factors such as affordability, availability, accessibility and acceptability [13], stigma remains a barrier to accessing and utilizing care by marginalized groups [14]. Further contributing to underutilization of oral care services are dentists who may feel uncomfortable treating certain individuals, and make excuses such as being too busy, or not having appointments available [15].

Shrivastava and colleagues have proposed to explore and better understand self-perceived stigma and its impact on healthcare-seeking behavior [16]. In order to explore the extent to which individuals with history of substance addiction and mental illness experience stigmatizing attitudes and behaviours from healthcare providers in general and of dental professionals in particular, we employed Link and Phelan’s conceptualization of stigma within a qualitative study. This research question was framed to highlight that the medical healthcare system in general is an inherent part of the daily lives of these individuals, and that their healthcare coverage likely includes dental care [5]. Because of that, we attempted to explore the dental care aspects of their lives in the realm of the healthcare as a whole.

## Methods

### Data collection

Approval for this research was obtained from the Behavioural Research Ethics Board at University of British Columbia (#H12-03176). We used purposive and convenience sampling to recruit participants from two community treatment centres in the Greater Vancouver area of British Columbia, Canada via posters on their bulletin boards. The two centres provide specialized multidisciplinary inpatient treatment services for adults over 19 years with severe and complex concurrent substance addiction and mental health disorders who need either acute or long-term care from assessment and stabilization to treatment and psychosocial rehabilitation. As per our inclusion criteria, participants who reported they had visited a dental office after being diagnosed with one or more types of mental illnesses and who had dependence on one or more substances were invited to participate. The study variables included gender, drug of choice and years of use, age, and type of mental illness. Participants were all English-speaking and had the Ministry of Health-Disability insurance which every two years provides recipients with one thousand dollars towards basic dental care treatment. The British Columbia Employment and Assistance for Persons with Disabilities Act defines a person with disabilities as an individual who is at least 18 years of age, with a severe physical or mental impairment, and who is significantly restricted in his or her ability to perform daily living activities, and/or who requires assistance with daily living activities. The dental insurance covers basic intra-oral examinations, radiographs and dental treatment including fillings, extractions and some limited prosthodontic procedures.

Table 1 shows the self-reported demographic information of the participants and their addiction and mental illness status (Table 1). No attempt was made to confirm or verify the self-reported information given by our participants with clinical data or medical charts; we took as accurate what participants reported given such trust is the underlying assumption of a qualitative study. We operated under the framework that each participant had his or her own reality that was true to them; each reality was valid and worth understanding [17].

**Table 1:** Self-reported characteristics of participants (N = 25).

Participant	Gender	Age	Mental Health Diagnosis	Substance use	Years of Use
1	Male	37	Depression, Anxiety, Mood Disorders	Alcohol	22
2	Female	39	Depression	Alcohol	20
3	Male	28	Depression	Heroin, Crack, Alcohol	8
4	Male	ND	Obsessive Compulsive Disorder	Alcohol	ND
5	Male	28	Depression, Schizophrenia	Methamphetamine	11
6	Male	53	Paranoia Schizophrenia	Crack, Cocaine	20
7	Female	43	Bipolar Disorder	Cocaine	4
8	Male	41	Bipolar Disorder, Anxiety, PTSD	Methamphetamine	18
9	Male	54	Anxiety, Depression	Heroin, Cocaine, Methamphetamine	10
10	Male	52	Bipolar Disorder	Cocaine	35
11	Female	35	Depression, Anxiety, ADHD	Cocaine	2
12	Male	37	PTSD	ND	ND
13	Male	26	Bipolar Disorder	ND	ND
14	Male	35	Anxiety, Mood Disorders	Heroin, Alcohol	12
15	Male	31	Depression	Alcohol	19
16	Male	23	Depression	Crack, Alcohol	5
17	Female	56	Obsessive Compulsive Disorder	Alcohol	4
18	Male	24	Schizophrenia	Methamphetamine	9
19	Male	28	Schizophrenia	Crack, Cocaine	6
20	Male	30	Bipolar Disorder	Crack, Cocaine	7
21	Female	40	Bipolar Disorder, PTSD, Anxiety	Methamphetamine	11
22	Male	ND	Anxiety, Depression	Heroin, Cocaine, Methamphetamine	19
23	Male	67	Bipolar Disorder	Cocaine	41
24	Female	39	Anxiety, Mood Disorders	Alcohol	10
25	Male	45	PTSD	Crack, Alcohol	12

ND- Not Disclosed; PTSD–Post-traumatic Stress Disorder; ADHD–Attention Deficit Hyperactivity Disorder

**Fig 1:**
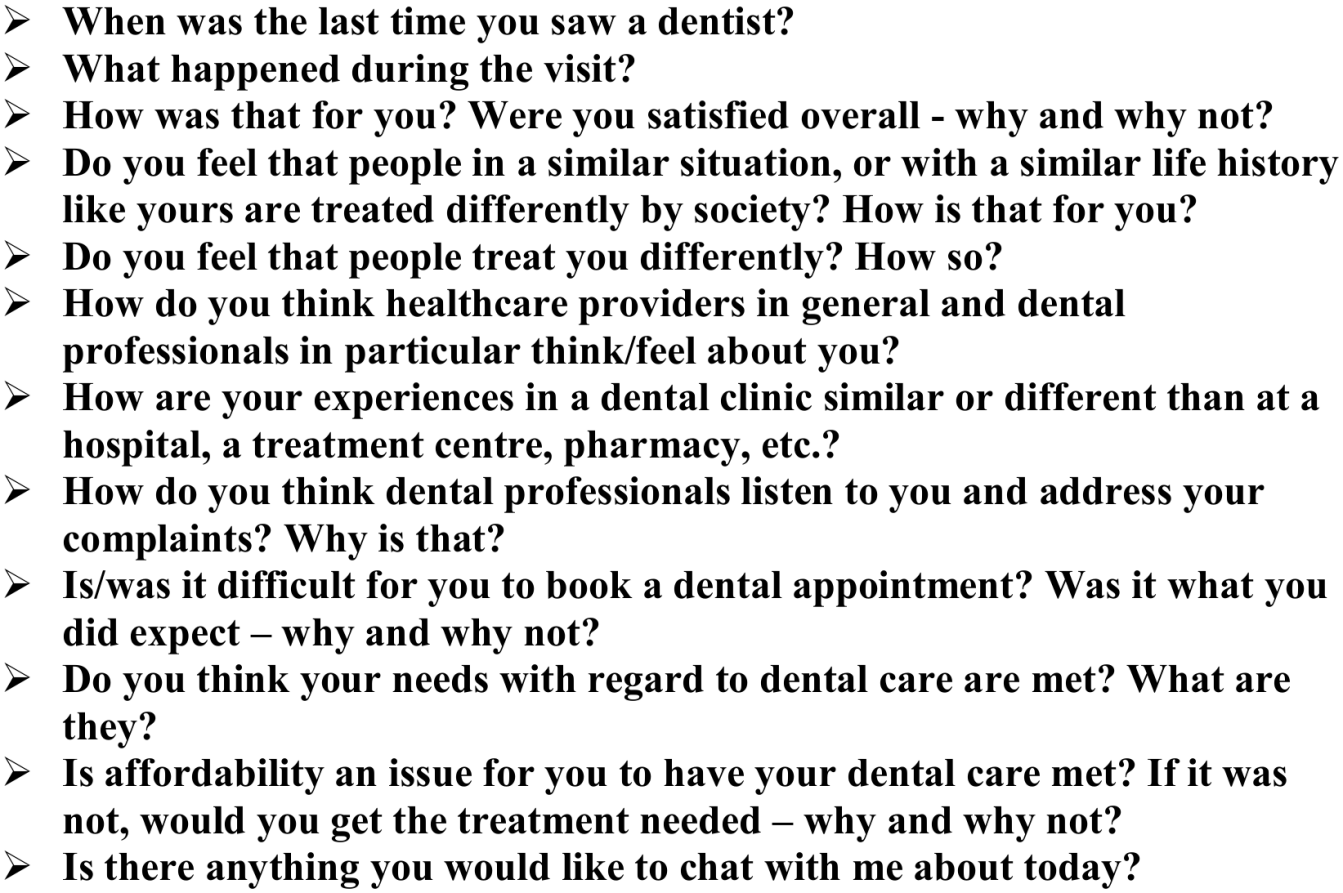
Interview guide.

After written consent was obtained and a brief demographic questionnaire was completed individually, personal interviews with open-ended questions based on Link and Phelan’s [2] model of stigma were employed so that we could explore how the different domains of the model related to the participants’ health and dental care experiences, if at all. Although other models of stigma exist [5], Link & Phelan is the only one to explicitly acknowledge the influence of power as well as the society and its structural organization in fostering or hindering the development of stigma; that was the preamble for our interview guide. All interviews were conducted once by one of the authors (RA) in private spaces at the centres where the participants resided at the time of the interview, and were audio-recorded. Participants were not directly asked if they felt stigmatized or labeled to avoid biasing their answers (Fig 1). For example, they were asked “how do you think healthcare providers in general and dental professionals in particular think/feel about you?” and “why did you think they feel that way?” instead of directly being asking “did you feel you were labeled; stereotyped; stigmatized; discriminated?” We opted for this approach since some individuals may not feel stigmatized or labeled per se and yet, they may have been treated differently in many ways. This rationale was used to avoid directing participants to say they were stigmatized just because we were asking them, and to explore the behaviors and attitudes they felt towards them. Short responses were managed by probing questions for more detailed information (e.g., “can you tell me more about that?” or “why did you feel that way?”). Participants were thanked for their time, and were given a $20 gift card to a local coffee shop, while personal hygiene products were purchased and donated to the two community treatment centres as requested by the centres’ staff.

We aimed to interview enough participants to reach saturation; a point in which no new information about stigma emerged and the content became repetitive yet with enough depth for the thematic analysis [18]. After conducting 25 interviews, we reached saturation of general information when no new obvious facts and experiences about stigma emerged, although concepts like this that can be broad may not have their understanding fully saturated. Each interview lasted 20 to 75 minutes. Some interviews were challenging due to the lack of engagement, difficulty maintaining a conversation or simply because participants had other priorities in their daily routine; some participants tired easily and we decided to not continue their interviews. Consequently, we found unfeasible and non-informative to conduct member checks to share the preliminary analysis with the participants despite our attempt to do with five of them.

### Data collection and data analysis

**Fig 2:**
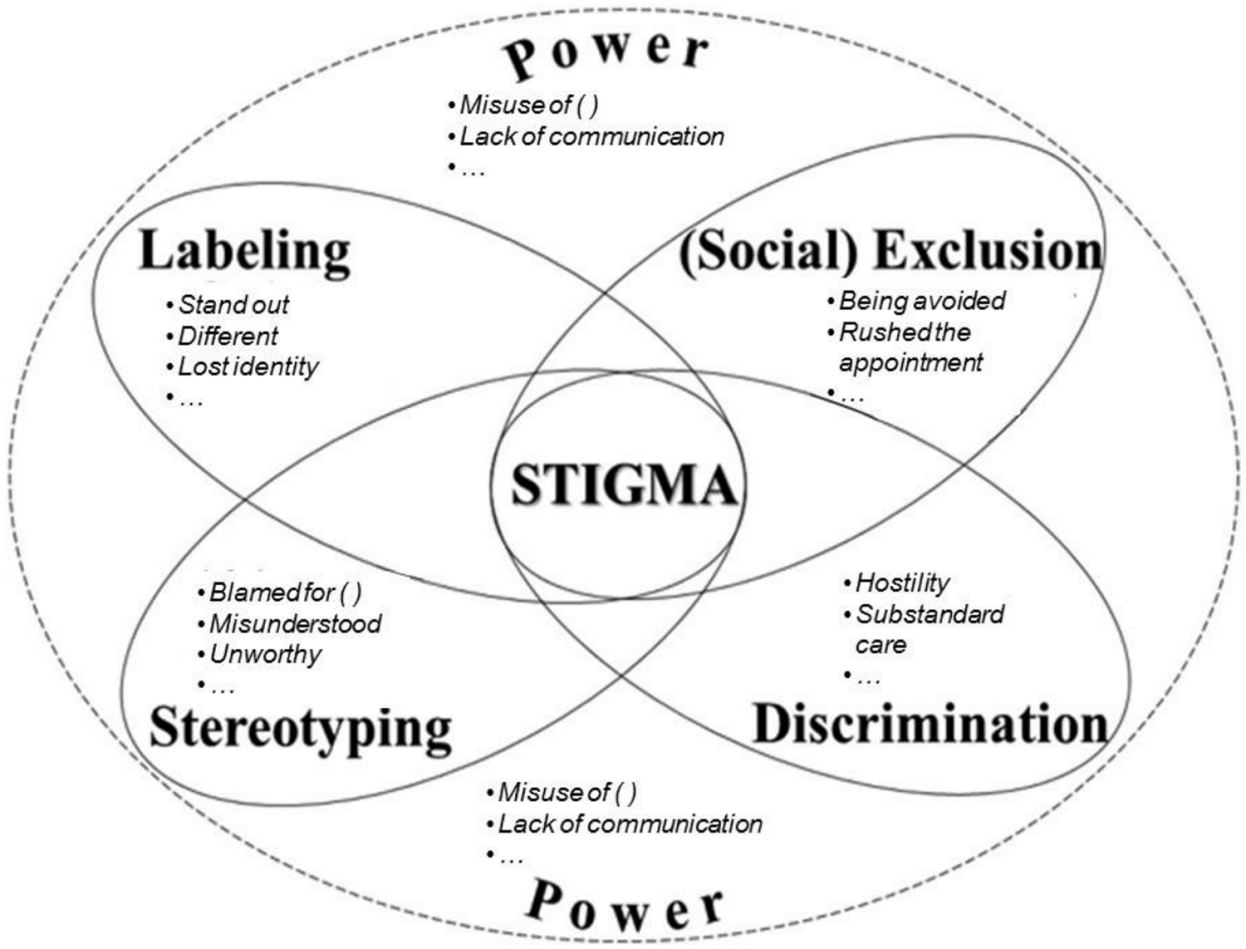
A visual representation of stigma (domains and their respective themes).

Data collection and data analysis occurred simultaneously so that we could identify the strengths and drawbacks of the interview guide (Fig 1) and introduce modifications for the subsequent interviews as needed. Following completion of each interview, one of the authors (RA) listened to the audio-recordings several times and transcribed all content *verbatim*. Within the first two transcripts, two authors (RA and MAB) individually attached a denomination (e.g., a code–usually a single word in the form of verb, noun or an adjective) to each couple of words or phrase(s) they judged important from what participants had mentioned. This interactive coding process helped the authors identify repeating ideas within and between transcripts which were then categorized as emerging themes even though an ‘idea’ may have appeared only once in only one transcript; it also enabled rigour of the study since an audit trail was used as two authors were coding the first two transcripts using similar denominations. One of the authors (RA) then carried out the remainder of the analysis that was brought back to the other authors for discussion. We attempted to group the themes under the domains of stigma from the model presented in Fig 2.

## Results

The transcribed interviews were made anonymous while retaining the essential demographic characteristics of each participant as per Table 1. The individual interviews revealed that participants experienced stigma in healthcare and dental settings both directly and indirectly. We present our findings under the pre-existing domains proposed by Link and Phelan^2^ while highlighting the main themes as underlined below and portrayed in Fig 2.

### Labeling

Participants reported that their struggles with addiction and mental illness had caused them to stand out from the norm, and be labeled as different. Participants were given names (e.g., labels) such as “*alcoholic*”, “*addict*”, “*pot/crack head*”, “*junkie*”, “*loser*”, and “*crazy*”. The majority of the participants mentioned these labels as being common on the streets but were seldom used by health professionals including dentists. However, they did believe that these professionals *“label [the patients] in their head*, *in their own time when talking amongst themselves*.*”*

Participants also believed they had directly or indirectly lost identity to their labels even for those who had already recovered from their addictions. For example, participant 19 mentioned “*[It’s like] you’re not yourself anymore*, *you’re the schizophrenic*, *or the crack-addicted*.” Furthermore, as healthcare providers have access to the patients’ medical records on substance use and/or mental illness, that seemed to trigger and practically serve as negative labels:

“*I went to [an] emergency room one time and the lady came back after checking my record and barely talking to me; she said ‘well there is nothing we can do*, *we can’t help you here’ and she didn’t even get to talk to me; she was going by my records*.”

(Participant 6)

For some participants, dental providers have less access to their full medical history and charts, which prompted participant 22 to mention *“if the dentist does not ask me specifically about that*, *I do not tell*. *It is not like I carry my medical chart with me anywhere I go…do they even have to know*?*”* It must be mentioned that participants whose symptoms of addiction or mental illness were not obvious or were under the radar did not lead us to believe they felt labeled: “*in the [dental] office I tried to*, *you know*, *act as normal as possible without raising my profile too much*” (Participant 13).

### Stereotyping

The majority of participants’ experiences signified one or more stereotypes that health professionals including dentists held against them. In fact, several participants described how some dentists appeared to blame them for their oral health status: “*They [dentists] think that I wrecked my teeth and I shouldn’t have done that*” (Participant 5). Such criticisms made participants feel misunderstood and not cared for. In particular, some participants thought it was unfair to be blamed for their poor oral health as they told us that they were never taught about how their life conditions could negatively impact the health of their mouths.

Participants also believed that health providers in general considered them inferior and unworthy, as explained by participant 2:

“*I think that [the health provider] did his job and everything but I think that he thinks less highly of me… I just know that he doesn’t think I was like a normal working*, *outstanding person*.”

Many participants talked about how dentists in particular seemed to think that not only were they unable to change but also unwilling to improve the health of their mouths and teeth. These participants asserted that the social and personal aspects of substance use and mental illness are often overlooked and that stigma at the level of stereotyping is consequent to ignorance of the healthcare professional about issues that typically surround the development of addiction and mental illness as voiced by participant 19: *“What would they [dentists] expect*? *That I would stop the fix [as in injecting the drug]*, *floss my teeth*, *and get high again*?…*that ain’t gonna happen*!

Another stereotype felt by our participants was untrustworthiness, especially from dentists when withholding pain medications: “*I had issues with pain in my mouth and when asked about painkillers*, *the [dentist] assumed I was seeking drugs*” (Participant 21). Lastly, some participants recognized that they were seen as dangerous and aggressive. Participant 12, for example, said that “*[people] are careful*, *they are scared and probably afraid of the craziness…the swing moods*.” In a dental appointment, participant 3 felt her current dentist appeared agitated when treating her: “*Sometimes [the dentist] looks as if he is afraid of me; he shakes*, *he seems to be nervous and I noticed that*.”

### (Social) Exclusion

Although it is unlikely that health professionals would purposely seek out social interactions with any of their patients, the feeling of being avoided was felt in the dental setting as we explain under discrimination ahead. Nonetheless, dental appointments happen in a social context and yet dentists seemed to minimize interactions at best or avoid altogether at worst without even making eye contact, some patients told us. Some other dentists, participants informed us, seemed to rush the appointment once they were informed or guessed about the substance use and mental illness: “*[the] dentist seemed to have performed the procedure too quickly*, *not waited long enough for anaesthetics to become effective*, *was impatient when explaining oral hygiene to me*, *and did not take the time to answer to the questions fully”* (Participant 14).

### Discrimination

Many of the participants’ experiences with discrimination involved being rejected by health professionals, particularly in hospitals and emergency rooms. In the case of dentistry, discrimination took form when they felt hostility from the dentists and dental team, or thought they received substandard care. We heard that dentists seemed to make excuses to indirectly reject patients and some of the reasons participants were given for being refused were related to their disability insurance:

“*The dentist did some work [then] said I have more work to do*. *But she told me to go to [another clinic]… She said it is cheaper and I will get more out of my money*.*”*

(Participant 6)

Another recurrent perceived experience that participants shared was healthcare professionals’ inattentiveness towards them. In dental settings in particular, participants felt neglected mostly when they sensed a lack of proper communication by not having their questions properly answered, for example. Besides ineffective communication, another set of experiences described by our participants was perceived unfair pain management (e.g., both provision of post-operatory pain medication and local anesthesia) as mentioned above. What made these experiences even harder for some participants was the fact that they did not feel they received proper acknowledgment of their discomfort. Participant 24 said, “*He never put enough freezing in it*…* it felt like he wasn’t even making sure I was ok*!”

The perception of substandard treatments, as a form of discrimination, also emerged from the interviews as many participants felt that the quality of healthcare that they were provided was poor. In dental settings, this was brought up mainly regarding the type of dental care provided. For instance, participant 3 who at the age of 28 only had ten teeth left in her mouth stated that “*[the dentist] said he would just pull the teeth now*. *He won’t bother having them fixed…*.” Another participant who was concerned about losing all his teeth also explained:

“*A lot of times [dentists] would just pull the tooth rather than work on it*, *rather than do what they have to do to save it… I’ve had two teeth ripped out like that*. *They could be saved to work on*, *but [the dentist] figured it was easier just to pull it*.”

(Participant 25)

### Power

Feelings of being labeled, stereotyped and discriminated against by health professionals lead our participants to also describe how they were powerless and did not have control over life situations. Misuse of power by health professionals in general was also brought up when participants talked about their experiences in healthcare settings. In the case of dental settings, lack of proper communication seemed to add to these feelings especially for patients who had dental anxiety, and became extremely stressed:

“*[The dentist] doesn’t always explain everything*, *and that makes me wonder*: *What are they doing*? *What are they putting in my mouth*? *What’s going on*? *It freaked me out*, *you know*!”

(Patient 17)

Moreover, many participants expressed feelings of being powerless and helpless when dentists (mis)used their power to withhold pain medications, as previously discussed. Participants’ solution to avoiding disempowerment was to cover up their conditions as much as possible, wishing to keep the information regarding their substance use private and hidden. Participants mentioned that they could not lie to the physicians who could find out about their condition through lab test results or medical records. However, as we presented when describing labeling above, participants believed that dentists were likely to not be informed about the same issues because:

“*[Doctors] would know more by checking around doing a blood test*, *or urine test so I would have to tell them the truth*, *but [the] dentist*, *he’s not gonna check into those kind of things so I don’t say anything…it is my private life*.”

(Participant 9)

### Positive experiences

Participants also described encounters with healthcare professionals including dentists that were characterized by care and understanding. When talking about the qualities of these professionals, participants used numerous terms including “*caring*, *nice*, *patient*, *courteous*, *gentle*, *kind*, *helpful*, *and fair*.*”* In dental settings in particular, they noticed when the dentist made eye contact, spoke in a pleasant voice, took the time to explain the procedure(s), tried to manage the pain and perhaps most importantly, did not look or talk down to them:

“*I like my dentist*. *I’m going to be moving onto transitional housing which is gonna be about 45 minute travel time and I plan on travelling back to see Dr*. *---*, *he’s excellent as he listens and bring me to the conversation*!”

(Participant 11)

All domains presented above as labeling, stereotyping, (social) exclusion, discriminating and disempowering, as well as most of the themes that were brought up and discussed during the interviews can be understood as linked to each other and interconnected (Fig 2, from Alan [5]). However, these domains do not need to be experienced altogether for stigma occur as represented by the four elliptical shapes in Fig 2. For example, a patient with addiction and mental illness could have been recognized as different and labeled “user” or “crazy”, and felt stigmatized. Others could have experienced being stereotyped and discriminated, but not (socially) excluded despite the entire experience disempowering them. As a result, many of the participants’ statements could be categorized under more than one theme; many themes and all domains should be seen as interconnected and overlapping with each other.

## Discussion

As Shrivastava and coworkers [16] have suggested to study stigma and its impact according to the individual, this qualitative research sought to explore how 25 purposefully selected interviewees with addiction and mental illness self-perceived stigma in the healthcare in general, and in dental settings in particular. Although not all themes that emerged from these 25 interviews have to be experienced by the same person for stigmatization to occur, the occurrence of one or a couple of themes presented herein can be detrimental to the well-being of anybody [14,15].

Our findings indicated that individuals with substance addiction and mental illness experience overt or subtle stigmatization in dental settings similarly to other healthcare settings and other groups (e.g., HIV positive, obese, prisoners, and prostitutes). For instance, like what was found by Puhl and Heuer [19] when interviewing individuals who are obese and by Brondani and colleagues [14] when working with patients who are HIV positive, our participants reported often being blamed for their poor health status and oral health problems. While it is common for dental professionals to criticize their patients for their poor oral hygiene and consequent dental diseases, Chandu (2011) highlighted that patients may perceive such criticisms as a lack of empathy and understanding [20]. The victim-blaming attitude is more problematic than beneficial; health professionals should take into account a patient’s life situation and environmental factors such as low level of knowledge or inability to access adequate resources without stereotyping them [21,22]. As advised by Bedos and Loignon (2011), a paternalistic approach lacks efficacy and is in direct contradiction to the more humanistic patient-centred care approach [23].

Many other misconceptions and stereotypes were reflected in our participants’ description of their experiences in health to a greater extent, and to a lesser yet detrimental extent in dental care settings. Most of these negative stereotypes were actualized in the health providers’ negative attitudes and the low quality of services perceived by patients. The perception of substandard dental treatment in particular in the form of extracting potentially salvageable teeth could be due to the dentist’s beliefs that patients with addiction and mental illness are unworthy, at fault, or have chosen their lifestyle, thus not deserving of complex and time-consuming treatment. It could be also that, due to limited financial resources by the patient and/or their dental plan, extensive treatment to salvage teeth would not be in the best interest of the patient. While this explanation is plausible, it should be noted that other stigmatized groups such as prisoners, prostitutes, patients who have attempted suicide, those living with HIV and the obese have received generally less effort from healthcare professionals [15,19,21,23,24,25]. Ultimately, to avoid misunderstandings, patients should always be well informed about prognosis and treatment possibilities and encouraged to take a more proactive role in the decision-making process. This helps also to shift positively the power relationship between provider and patient.

While medication being withheld was frequently mentioned during the interviews, it is important to keep in mind the global prescription drug crisis that may affect both stigmatized and non-stigmatized individuals. Canada, for example, has climbed from sixth to second largest prescription-opioid consumer in the world over the last decade; in 2013 the Canadian Centre on Substance Abuse reported that prescribed painkillers were the most dangerous drugs after tobacco and alcohol [26]. Dentists play a prominent role in addressing this health concern and preventing such abuse and deaths, particularly of opioids, as seen recently in major Canadian cities. As there is a call for change in medication prescription behavior, the impact may be more substantial for stigmatized groups such as those featured in this study especially given their history of substance abuse.

Although there is evidence that certain characteristics of patients may interfere with the normal routine of treatment, including uncooperativeness, lower social class, fear or anxiety, and mental disability [27], refusing to provide care remains unethical and unprofessional [28]. But irrespective of the patient’s characteristics, government-regulated plans are not welcomed by many dental clinics due to payment schemes and a lower fee reimbursement; therefore our participants may have experienced refusal of care as also found by others [29,30].

The stress and anxiety our participants expressed in terms of dental treatment perhaps exacerbated even more feelings of not having control of their situation, as discussed by Norberg and Boman [29]. Therefore, in order to help patients cope with their decreased sense of power, emphasis needs to be placed on communication, empathy and rapport building skills [22,30] as well as actively involving them in a shared decision-making process. In fact, participants’ positive experiences seemed to have occurred because dentists showed respect and compassion through effective communication, which may have led to an acceptable balance in the power relationship [5]. Nonetheless, the elliptical illustration presented in Fig 2 shows that, in the case of this study, the experience of stigma takes places within an imbalance of power within one or more of the following circumstances: being labeled, discriminated against, stereotyped or excluded.

Mental illness and addiction have negative implications for systemic and oral health and when associated with stigma, they further impact access to care. We suggest to continue to build on social awareness of health issues among current and future healthcare professionals to help improve the experiences for this marginalized population who already face numerous social, financial and other barriers in life [31]. Reducing oral health inequities is a matter of social justice, and dental care providers are key actors in this endeavour. Although this study intentionally did not include the dental professionals, our participants echoed what other studies have found: that there is a constant need to offer venues for these professionals to enhance social awareness and discuss how to create a safe environment for patient care regardless of one’s life conditions. Hence, further studies are needed to include a larger sample of similar patients to unravel their experiences and interactions with other health professionals and their staff, as well as the professionals’ own perspectives on the issues discussed herein. More research is also needed to better inform strategies for reducing stigma and increasing quality of care, particularly to those who feel stigmatized. However, increased social awareness and knowledge are not sufficient for stigma reduction and, therefore, further training on trauma informed care and culturally sensitive approaches are needed so that healthcare providers can appropriately work with stigmatized groups [13,32,33,34].

## Limitations

Our limitations included: generalizability is limited due to the relatively small sample size and because the characteristics of our participants may not be typical of the population with addiction and mental illness in Canada or elsewhere; in addition to substance use and mental illness, almost all participants had other stigmatizing characteristics (e.g., being poor, being of First Nations descent) so the stigma felt could have been a response to a combination of all these conditions; some experiences were not specific to dental settings, that is, the participants may have not necessarily distinguished between dental care providers and other healthcare providers when being interviewed about stigmatization; dental professionals were not included so their views on stigma of addictions and mental illnesses, although valuable, could not be compared to the community members we interviewed; although widely utilized, the conceptual model offered by Link and Phelan may have offered a limited and negative view of stigma; the auditing trail we used for the coding process was done using the first two transcripts only and because of that it may have introduced personal biases as one author alone coded the remainder of the interviews although the authors met frequently to discuss the coding and analysis.

## Conclusions

This study set out to explore how individuals with a history of addiction and mental illness perceived stigmatizing behaviours in healthcare settings including dental offices, either overt or subtle. We discovered that our participants perceived stigma when they were negatively stereotyped as ‘unworthy’, labeled as ‘different’, excluded from the decision-making process, discriminated against, ‘treated unfairly’, and felt powerless when interacting in the heath and dental care system. Conversely, positive experiences were characterized by empathy, reassurance and shared decision-making, which were empowering for patients. Our findings highlight the need for increased social awareness regarding the domains of stigma that we have presented among current and future health and dental care professionals in order to address the health inequities faced by individuals with addictions and mental illness.
